# First Report of the Potential Bovine Spongiform Encephalopathy (BSE)-Related Somatic Mutation E211K of the Prion Protein Gene (*PRNP*) in Cattle

**DOI:** 10.3390/ijms21124246

**Published:** 2020-06-15

**Authors:** Sae-Young Won, Yong-Chan Kim, Byung-Hoon Jeong

**Affiliations:** 1 Korea Zoonosis Research Institute, Jeonbuk National University, Iksan, Jeonbuk 54531, Korea; gkfh32@jbnu.ac.kr (S.-Y.W.); kych@jbnu.ac.kr (Y.-C.K.); 2 Department of Bioactive Material Sciences and Institute for Molecular Biology and Genetics, Jeonbuk National University, Jeonju, Jeonbuk 54896, Korea

**Keywords:** E211K, E200K, bovine spongiform encephalopathy, prion, somatic mutation, prion protein gene (*PRNP*)

## Abstract

Bovine spongiform encephalopathy (BSE) is a prion disease characterized by spongiform degeneration and astrocytosis in the brain. Unlike classical BSE, which is caused by prion-disease-contaminated meat and bone meal, the cause of atypical BSE has not been determined. Since previous studies have reported that the somatic mutation in the human prion protein gene (*PRNP*) has been linked to human prion disease, the somatic mutation of the *PRNP* gene was presumed to be one cause of prion disease. However, to the best of our knowledge, the somatic mutation of this gene in cattle has not been investigated to date. We investigated somatic mutations in a total of 58 samples, including peripheral blood; brain tissue including the medulla oblongata, cerebellum, cortex, and thalamus; and skin tissue in 20 individuals from each breed using pyrosequencing. In addition, we estimated the deleterious effect of the K211 somatic mutation on bovine prion protein by in silico evaluation tools, including PolyPhen-2 and PANTHER. We found a high rate of K211 somatic mutations of the bovine *PRNP* gene in the medulla oblongata of three Holsteins (10% ± 4.4%, 28% ± 2%, and 19.55% ± 3.1%). In addition, in silico programs showed that the K211 somatic mutation was damaging. To the best of our knowledge, this study is the first to investigate K211 somatic mutations of the bovine *PRNP* gene that are associated with potential BSE progression.

## 1. Introduction

Bovine spongiform encephalopathy (BSE) is a well-known prion disease characterized by the accumulation of abnormal prion protein (PrP^Sc^), which shows resistance to proteinase K [1,2,3,4,5,6,7,8,9,10,11,12,13]. Classical BSE was first reported in the United Kingdom (UK) in 1986 and has been propagated by PrP^Sc^-contaminated meat and bone meal. Since being controlled by more thorough surveillance of contaminated feed, the number of classical BSE cases has dramatically decreased [14,15]. However, novel forms of BSE and atypical BSEs have been reported in many countries. Unlike classical BSE, the cause of atypical BSE has not been determined [16,17,18].

In humans, prion diseases are classified as sporadic, familial, and infectious forms. Approximately 85% of all human prion diseases are sporadic Creutzfeldt–Jakob disease (CJD). Approximately 15% of all human prion diseases, including familial CJD, Gerstmann–Straussler–Scheinker syndrome (GSS), and fatal familial insomnia (FFI), are known to be caused by a germline mutation of the prion protein gene (*PRNP*) [19,20]. Additionally, <1% of all human prion diseases, including iatrogenic CJD, variant CJD, and kuru, are acquired by transmission. However, accurate causes of sporadic CJD have not been investigated to date [21,22]. Somatic mutations of the *PRNP* gene have been reported in the blood and brain tissue of sporadic cases of prion diseases. D178N and P102L somatic mutations have been reported in sporadic CJD patients and sporadic GSS symptom patients, respectively [23,24]. The commonality of these patients is that they were caused by somatic mutations, and not germline mutations inherited from their parents. Thus, somatic mutation of the *PRNP* gene was assumed to be one cause of sporadic prion diseases, in which no cause has been identified to date. In cattle, the E211K mutation of the bovine *PRNP* gene, which is homologous to the E200K mutation of the human *PRNP* gene found in familial CJD, was first reported in atypical BSE in the United States in 2006 [25,26,27]. At present, since atypical BSEs, which may refer to sporadically occurring BSE, have been occurring steadily in many countries, it is important to investigate the somatic mutation on E211K of the *PRNP* gene in cattle.

In this study, we investigated the E211K somatic mutation of the *PRNP* gene in Korean cattle using pyrosequencing. We collected peripheral whole blood and several types of tissue in cattle, including the medulla oblongata, cerebellum, cortex, thalamus, and skin tissue. We purified the genomic DNA from each tissue and blood and performed pyrosequencing to identify the E211K somatic mutation of the bovine *PRNP* gene. Furthermore, we estimated a deleterious effect of the E211K somatic mutation on bovine prion protein by in silico evaluation tools such as PolyPhen-2 and PANTHER [28,29].

## 2. Results

### 2.1. Sample Preparation

We collected samples from peripheral blood and five tissues, including the medulla oblongata, cerebellum, cortex, thalamus, and skin. Detailed information on the collected samples is described in Table 1. DNA extraction was performed with peripheral blood and brain tissues, and all DNA samples were subjected to quality checks using the A260/A280 ratio with 1.8–2.0 values.

### 2.2. Validation of the Inspection Tool

**Figure 1:**
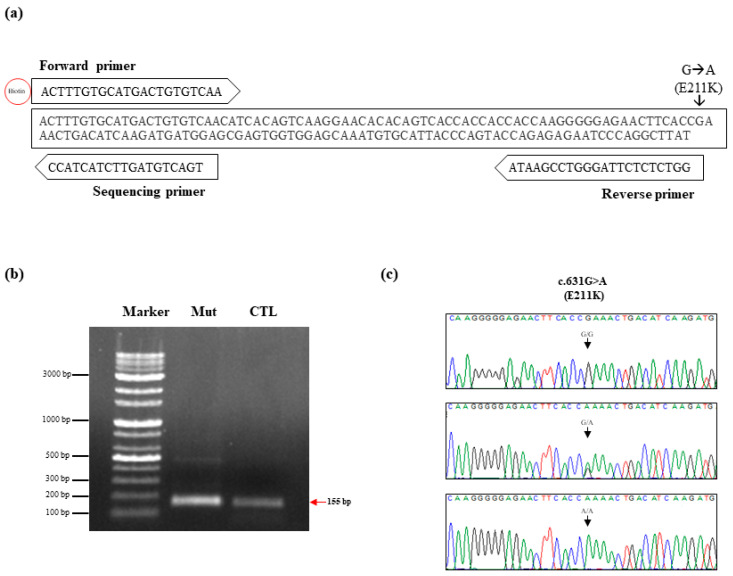
(**a**) Schematic design of pyrosequencing to detect the E211K somatic mutation of the bovine prion protein gene (*PRNP*). (**b**) Gene-specific primers were used to obtain 155 bp amplicons of the bovine *PRNP* gene containing K211 mutation and E211 wild type. Marker: 100 bp DNA ladder marker; Mut: K211 mutation sample; CTL: E211 normal sample. (**c**) Electropherograms of Sanger sequencing results. The upper panel indicates Sanger sequencing results of the DNA amplicon with 100% E211 normal sample. The middle panel indicates Sanger sequencing results of mixed DNA amplicons with 50% E211 normal sample and 50% K211 mutation sample. The lower panel indicates Sanger sequencing results of DNA amplicon with 100% K211 mutation sample.

**Figure 2:**
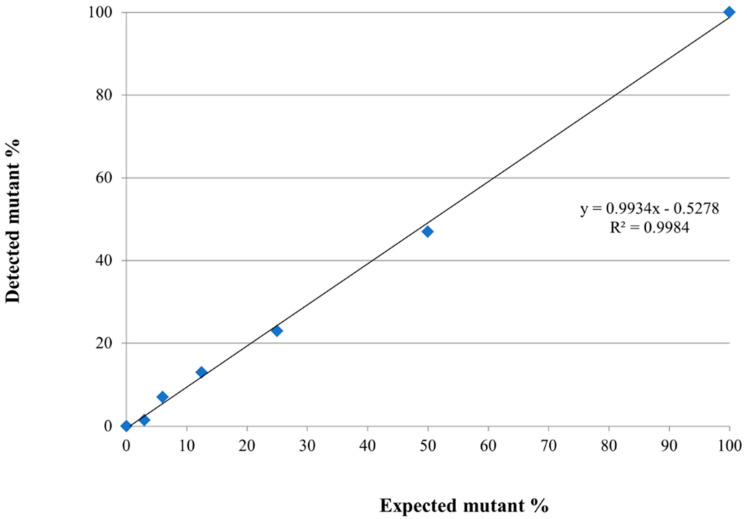
Validation of the detection accuracies of pyrosequencing. The scatter plot indicates the average of the observed somatic mutation rates of the standard materials with mutation rates of 100%, 50%, 25%, 12.5%, 6%, 3%, and 0%. Regression analysis was performed to determine the correlation between the observed somatic mutation rates and the expected somatic mutation rates.

To investigate the somatic mutation of K211 in the bovine *PRNP* gene, we performed pyrosequencing analysis, which is known as a convenient tool to measure somatic mutations with low frequencies. Detailed information is described in Figure 1a. The polymerase chain reaction (PCR) products containing the mutation site at codon 211 of the bovine *PRNP* gene were amplified using biotinylated forward and non-biotinylated reverse primers (Table 2). The pyrosequencing primer set (forward, reverse, and sequencing primer) was generated using PyroMark Assay Design (QIAGEN, Germany). To verify the accuracy of the detection of pyrosequencing, we synthesized the K211 mutant gene using PCR-based site-directed mutagenesis. Biotinylated PCR products were confirmed on a gel with a length of 155 bp (Figure 1b). Before the pyrosequencing analysis, we confirmed the K211 mutation of the standard material by Sanger sequencing (Figure 1c). Then, we mixed standard material of the E211 normal gene with that of K211 mutant genes at specific ratios. According to the manufacturer’s recommendation, reliable detection and quantification of sequence variation using pyrosequencing is above 5% mutation. Thus, we excluded the dilutions of extremely low levels (0.1–3%). Each experiment was performed in duplicate. The mutation rate, which was observed using pyrosequencing, showed a high correlation with the expected mutation rate (*R*^2^ = 0.9984, Figure 2).

### 2.3. Investigation of K211 Somatic Mutation of Bovine PRNP Gene in Korean Cattle

**Figure 3:**
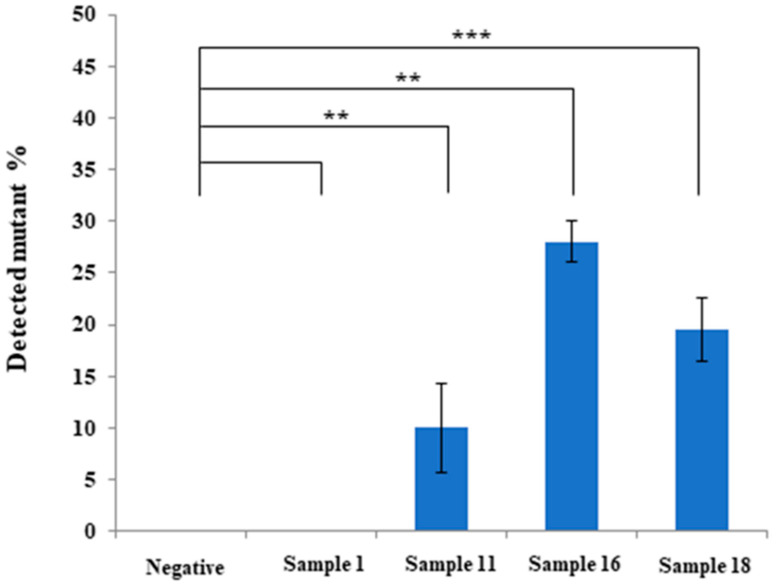
Representative results on the K211 somatic mutation rates of the bovine prion protein gene (*PRNP*) using pyrosequencing. Negative: standard material with 0% somatic mutation rates; Sample 1: Hanwoo, medulla oblongata; Sample 11: Holstein, medulla oblongata; Sample 16: Holstein, medulla oblongata; Sample 18: Holstein, medulla oblongata. All experiments were repeated three times. ** indicates *p* < 0.01 and *** indicates *p* < 0.001.

We performed pyrosequencing to examine the K211 somatic mutation of the *PRNP* gene in 58 brain and blood samples of Hanwoo and Holstein cattle. Detailed information on the pyrosequencing results is shown in Table 3. Among the 58 samples, three samples of the medulla oblongata of Holstein cattle, including samples 11, 16, and 18, showed somatic mutation rates of >10% (Table 3). The comparison of detection results between 0% negative standard and four bovine samples was analyzed using the Student’s *t*-test. Notably, samples 11 (10% ± 4.4%), 16 (28% ± 2%), and 18 (19.55% ± 3.1%) showed significantly high K211 somatic mutation rates (Table 3 and Figure 3).

### 2.4. In Silico Estimation of the Deleterious Effect of K211 Somatic Mutation of Bovine Prion Protein

To validate the prediction effectiveness of the annotation software, prior to the analysis of the K211 somatic mutation of the bovine *PRNP* gene, we analyzed the previously reported pathogenic *PRNP* mutations related to human genetic prion diseases. Although six mutations of human *PRNP* (D167N, V180I, V203I, I215V, M232R, and M232T) were predicted to be deleterious by only one program, the remaining 34 pathogenic mutations were predicted to be deleterious by PolyPhen-2 and PANTHER (Supplementary Table S1). We estimated the biological impact of the somatic mutation c.631G>A (E211K) of the bovine *PRNP* gene using in silico programs, including PolyPhen-2 and PANTHER. PolyPhen-2 and PANTHER predicted that E211K was “probably damaging” with a score of 0.993 and “possibly damaging” with a score of 361, respectively (Table 4).

## 3. Discussion

Although the number of classical BSE cases caused by contaminated meat and bone meal has decreased dramatically due to global efforts, the number of atypical BSE cases increased [30,31]. In humans, genetic prion disease makes up approximately 10% to 15% of cases with germline mutations of the *PRNP* gene [19]. However, although sporadic prion disease accounts for 85% of human prion diseases, the exact cause of sporadic prion disease has not been revealed to date. In recent studies, somatic mutations in the *PRNP* gene have been identified in human sporadic prion diseases and have been suggested as one cause of sporadic prion disease. In cattle, the E211K germline mutation of the bovine *PRNP* gene was first reported in the United States in 2006 [25,26,27]. The bovine E211K mutation showed homology in the region with the E200K mutation of the human *PRNP* gene, which is most frequently observed in human familial prion diseases [20,22]. Thus, we investigated the E211K somatic mutation of the bovine *PRNP* gene, which may be considered a novel risk factor for BSE in Korean cattle.

In our previous study, the germline mutation at codon 211 of the bovine *PRNP* gene was not observed in 384 Hanwoo cattle and 152 Holstein cattle [5]. Remarkably, we found high rates of K211 somatic mutation of the bovine *PRNP* gene in three Holstein cattle (Table 3). In Korea, since classical and atypical BSEs have never been reported in Hanwoo, the absence of K211 somatic mutations in Hanwoo is notable. In addition, although we tested K211 somatic mutations, including whole blood and four brain regions, somatic mutations of this gene were detected only in the medulla oblongata. The expected level of K211 mutations of the bovine *PRNP* gene, which can initiate atypical BSE, is elusive. A previous study in early-onset Alzheimer’s disease reported that 14% of mutations of the presenilin 1 (*PSEN1*) gene in brain cells are responsible for the initiation of this disease [32]. In another study, the somatic mutations of amyloid precursor protein (*APP*), nicastrin (*NCSTN*), sortilin-related receptor (*SORL1*), and microtubule affinity-regulating kinase 4 (*MARK4*) were observed in sporadic Alzheimer’s disease patients in 0.2–10.5% [33]. We observed three samples containing over 10% K211 mutations (Figure 3). Thus, BSE inspection of these samples seems essential to elucidate the expected level of K211 mutations of the *PRNP* gene, which can initiate atypical BSE in the future.

According to previous studies, since the medulla oblongata showed a prominent accumulation of PrP^Sc^, the diagnosis of BSE was performed in the medulla oblongata region [34]. However, since the three Holstein cattle carrying the K211 somatic mutation in the medulla oblongata were not investigated as to whether K211 somatic mutation could be observed in other brain regions, it is difficult to conclude that this somatic mutation was only found in the medulla oblongata. In addition, since atypical BSE has shown to be a prominent brain pathology in the frontal cortex [17,18], if the atypical BSE was caused by a somatic mutation of the *PRNP* gene, it is expected that the somatic mutation was also found in the cerebral cortex. Thus, further investigation of the K211 somatic mutation is highly desirable in other brain regions, including the cerebral cortex of the three Holstein cattle carrying K211 somatic mutation in the medulla oblongata. We also performed in silico analysis using PolyPhen-2 and PANTHER to evaluate whether the mutation of K211 of the bovine *PRNP* gene affects the bovine PrP protein. Notably, the K211 somatic mutation of the bovine *PRNP* gene was evaluated as “damaging”. Since PolyPhen-2 can estimate structural variation, the prediction can be interpreted that K211 somatic mutation can contribute to the conformational change to the susceptible structure of the PrP^Sc^. In addition, the wild-type PrP with E211 allele was predicted to be of high preservation time (361, Table 4) and K211 somatic mutation was estimated to be deleterious by using the PANTHER program. The prediction of PANTHER indicated that K211 mutation has been very rare and has not been observed in ancestral proteins for a long time. To confirm the effect of K211 mutation on prion disease, investigation of symptoms of prion disease in bovine PrP transgenic mice carrying the K211 allele is highly desirable in the future. It seems possible that a high somatic mutation rate contributes to sporadic prion disease in cattle.

Previous studies have reported that prion disease is accelerated in transgenic (Tg) mice expressing wild-type bank vole prion protein (BVPrP) containing E200K, which is associated with human familial CJD [35]. In addition, recent studies have demonstrated that a human PrP Tg mouse model with the E200K mutation can lead to spontaneous prion disease [36]. In cattle, the E211K mutation, which is a homologous region of human E200K, is expected to have a deleterious effect on BSE, and several in silico programs in the present study are expected to cause structural changes in the bovine prion protein (Table 4). To determine whether the E211K mutation affects the susceptibility to prion disease, brain samples with high mutation rates of E211K should be investigated by protein misfolding cyclic amplification (PMCA) or Western blotting (WB) in the future.

## 4. Materials and Methods

### 4.1. Ethics Statement

Peripheral whole blood, brain, and skin tissues were collected from slaughterhouses in the Republic of Korea. All experimental procedures were approved by the Institute of Animal Care and Use Committee of Jeonbuk National University (JBNU 2018-079) and approved on 1 October 2018. All experiments using cattle were performed following the Korean Experimental Animal Protection Act.

### 4.2. Genomic DNA Extraction

Genomic DNA was extracted from 20 mg of tissue using a Tissue Genomic DNA Isolation kit (QIAGEN, Germantown, MD, USA) and 200 µL of blood using a Blood Genomic DNA Isolation kit (QIAGEN, USA) following the manufacturer’s instructions.

### 4.3. PCR 

PCR was performed using *Taq* DNA Polymerase (QIAGEN, Hilden, Germany) following the manufacturer’s instructions. The PCR mixture contained 20 pmol of each primer, 5 µL of 10× *Taq* DNA polymerase buffer, 1 μL of 10 mM dNTP mixture, and 2.5 units of *Taq* DNA polymerase. The PCR conditions for each primer are described in Table 1. PCR was performed using an S-1000 Thermal Cycler (Bio-Rad, Hercules, CA, USA).

### 4.4. Site-Directed Mutagenesis

PCR-based site-directed mutagenesis was performed to obtain a DNA fragment of mutant type K211 of the bovine *PRNP* gene, which was used as a positive control. Briefly, 4 oligonucleotide primers were synthesized to carry out site-directed mutagenesis. Detailed information on the primers and experimental conditions is described in Table 1. In the first round of PCR, each pair (F1 pair and F2 pair) was amplified from a targeted region of the bovine *PRNP* gene. The second round of PCR was performed by F1_F and F2_R primer pairs, and amplicons amplified by the F1 pair and F2 pair were used as templates to obtain a DNA fragment of mutant K211 of the bovine *PRNP* gene.

### 4.5. Sanger Sequencing

The PCR products were purified using a PCR Purification Kit (Thermo Fisher Scientific, Bridgewater, NJ, USA) and directly analyzed with an ABI 3730 automatic sequencer (ABI, Foster City, CA, USA). Sequencing results were read by Finch TV software (Geospiza Inc., Seattle, WA, USA).

### 4.6. Pyrosequencing

Pyrosequencing was performed using biotinylated amplicons of the bovine *PRNP* gene by PCR. Briefly, the genomic DNA (10 ng) isolated from cattle was amplified by PCR using a biotinylated forward primer and a non-biotinylated reverse primer. Detailed information on the primers and PCR conditions is described in Table 1. The PCR products (25 µL) were mixed with 2 µL Streptavidin Sepharose High-Performance medium (GE Healthcare, Madison, WI, USA), 40 µL PyroMark Binding Buffer (QIAGEN, USA), and 8.5 µL high-purity autoclaved water, and centrifuged for 15 min at 14,000 rpm. The resulting products were washed with 70% ethanol for 10 s, PyroMark denaturation buffer (QIAGEN, USA) for 10 s, and PyroMark wash buffer (QIAGEN, USA) for 10 s, and then mixed with 24.2 µL PyroMark annealing buffer (QIAGEN, USA) and 0.8 µL sequencing primer (2 µM). Finally, the prepared samples were loaded into the PyroMark Q24 (QIAGEN, USA) and operated in the allele quantification (AQ) mode according to the manufacturer’s protocol.

### 4.7. Statistical Analysis

Three independent experiments were carried out, and the data were reported as the mean values ± standard deviation (SD). Statistical significance using the *p*-value was calculated with SAS version 9.4 (SAS Institute Inc., Cary, NC, USA) using a two-tailed Student’s *t*-test for single comparisons. The symbols **, and *** indicate *p* < 0.01, and *p* < 0.001, respectively.

### 4.8. In Silico Estimation of the Impact of E211K Somatic Mutation of the Bovine Prion Protein

A deleterious effect of somatic mutation E211K in bovine prion protein was evaluated by the PolyPhen-2 (http://genetics.bwh.harvard.edu/pph2/index.shtml) and PANTHER (http://www.pantherdb.org/) programs. PolyPhen-2 analyzed the impact of amino acid substitution on the protein of interest based on various features, including the functional or binding properties of sequence, phylogenetic property, and 3D structural information characterizing the substitution. PANTHER predicted the likelihood of a specific amino acid substitution in causing a functional impact on the protein by calculating the length of time that a given amino acid has been evolutionarily preserved to the protein of interest. The length of the preservation time is proportional to the magnitude of the likelihood of the functional impact.

## 5. Conclusions

In this study, we first identified a total of three high somatic mutation rates of E211K in three Holsteins using pyrosequencing. Next, we performed in silico estimation of the E211K mutation of the bovine *PRNP* gene using PolyPhen-2 and PANTHER, and it was predicted to be damaging. To the best of our knowledge, this study was the first to investigate potential BSE-associated K211 somatic mutations of the bovine *PRNP* gene.

## Figures and Tables

**Table 1:** Detailed information of the bovine samples used.

Inspection Site		Sex	Hanwoo	Holstein
Brain	Medulla oblongata	Male	5	5
		Female	5	5
	Cerebellum	Male		3
		Female	3	
	Cortex	Male		3
		Female	3	
	Thalamus	Male		3
		Female	3	
Peripheral blood		NA	5	5
Skin tissue		NA	5	5

NA: not available.

**Table 2:** Detailed information on the primer set used.

Purpose		Name	Sequence	Size	Annealing Temperature
Site-directed mutagenesis	Round 1	F1_F	ATGGTGAAAAGCCACATAGGCAG	652 bp	55 °C
		F1_R	CCATCATCTTGATGTCAGTTTtGGTGAAGTTCTC		
	Round 1	F2_F	GAGAACTTCACCaAAACTGACATCAAGATGATGG	204 bp	55 °C
		F2_R	GATAATGAAAACAGGAAGGTTGCCCC		
	Round 2	F1_F	ATGGTGAAAAGCCACATAGGCAG	820 bp	55 °C
		F2_R	GATAATGAAAACAGGAAGGTTGCCCC		
Pyrosequencing	Amplification	Biotin_PF	ACTTTGTGCATGACTGTGTCAA	155 bp	58 °C
		PR	ATAAGCCTGGGATTCTCTCTGG		
	Sequencing	Seq_R	CCATCATCTTGATGTCAGT		

**Table 3:** K211 somatic mutation rates of the prion protein gene (*PRNP*) observed in Hanwoo and Holstein cattle.

Sample No.	Breeds	Sex	Inspection Site	* AVG of Detected Mut. (%)	** STD
Sample 1	Hanwoo	Female	Medulla oblongata	0	0
Sample 2	Hanwoo	Female	Medulla oblongata	0	0
Sample 3	Hanwoo	Female	Medulla oblongata	0	0
Sample 4	Hanwoo	Female	Medulla oblongata	0	0
Sample 5	Hanwoo	Female	Medulla oblongata	0	0
Sample 6	Hanwoo	Male	Medulla oblongata	0	0
Sample 7	Hanwoo	Male	Medulla oblongata	0	0
Sample 8	Hanwoo	Male	Medulla oblongata	0	0
Sample 9	Hanwoo	Male	Medulla oblongata	0	0
Sample 10	Hanwoo	Male	Medulla oblongata	0	0
Sample 11	Holstein	Female	Medulla oblongata	10	4.4
Sample 12	Holstein	Female	Medulla oblongata	0	0
Sample 13	Holstein	Female	Medulla oblongata	0	0
Sample 14	Holstein	Female	Medulla oblongata	0	0
Sample 15	Holstein	Female	Medulla oblongata	0	0
Sample 16	Holstein	Male	Medulla oblongata	28	2
Sample 17	Holstein	Male	Medulla oblongata	0	0
Sample 18	Holstein	Male	Medulla oblongata	19.55	3.1
Sample 19	Holstein	Male	Medulla oblongata	0	0
Sample 20	Holstein	Male	Medulla oblongata	0	0
Sample 21	Hanwoo	Female	Cerebellum	0	0
Sample 22	Hanwoo	Female	Cerebellum	0	0
Sample 23	Hanwoo	Female	Cerebellum	0	0
Sample 24	Holstein	Male	Cerebellum	0	0
Sample 25	Holstein	Male	Cerebellum	0	0
Sample 26	Holstein	Male	Cerebellum	0	0
Sample 27	Hanwoo	Female	Cortex	0	0
Sample 28	Hanwoo	Female	Cortex	0	0
Sample 29	Hanwoo	Female	Cortex	0	0
Sample 30	Holstein	Male	Cortex	0	0
Sample 31	Holstein	Male	Cortex	0	0
Sample 32	Holstein	Male	Cortex	0	0
Sample 33	Hanwoo	Female	Thalamus	0	0
Sample 34	Hanwoo	Female	Thalamus	0	0
Sample 35	Hanwoo	Female	Thalamus	0	0
Sample 36	Holstein	Male	Thalamus	0	0
Sample 37	Holstein	Male	Thalamus	0	0
Sample 38	Holstein	Male	Thalamus	0	0
Sample 39	Hanwoo	NA	Peripheral blood	0	0
Sample 40	Hanwoo	NA	Peripheral blood	0	0
Sample 41	Hanwoo	NA	Peripheral blood	0	0
Sample 42	Hanwoo	NA	Peripheral blood	0	0
Sample 43	Hanwoo	NA	Peripheral blood	0	0
Sample 44	Holstein	NA	Peripheral blood	0	0
Sample 45	Holstein	NA	Peripheral blood	0	0
Sample 46	Holstein	NA	Peripheral blood	0	0
Sample 47	Holstein	NA	Peripheral blood	0	0
Sample 48	Holstein	NA	Peripheral blood	0	0
Sample 49	Hanwoo	NA	Skin tissue	0	0
Sample 50	Hanwoo	NA	Skin tissue	0	0
Sample 51	Hanwoo	NA	Skin tissue	0	0
Sample 52	Hanwoo	NA	Skin tissue	0	0
Sample 53	Hanwoo	NA	Skin tissue	0	0
Sample 54	Holstein	NA	Skin tissue	0	0
Sample 55	Holstein	NA	Skin tissue	0	0
Sample 56	Holstein	NA	Skin tissue	0	0
Sample 57	Holstein	NA	Skin tissue	0	0
Sample 58	Holstein	NA	Skin tissue	0	0

* AVG of detected mut. (%): average of detected percentage of K211 mutations; ** STD: standard deviation; NA: not available.

**Table 4:** In silico evaluation of the impact of K211 somatic mutation on the bovine prion protein.

Somatic Mutation	Methods	Score	Prediction
c.631G > A (E211K)	PolyPhen-2	0.993	Probably damaging
	PANTHER	361	Possibly damaging

## Supplementary Materials

Supplementary materials can be found at https://www.mdpi.com/1422-0067/21/12/4246/s1.

## Abbreviations

APP	amyloid precursor protein
AQ	allele quantification
BSE	bovine spongiform encephalopathy
BVPrP	bank vole prion protein
PrP^Sc^	abnormal prion protein
UK	United Kingdom
CJD	Creutzfeldt–Jakob disease
GSS	Gerstmann–Straussler–Scheinker syndrome
FFI	fatal familial insomnia
*MARK4*	microtubule affinity-regulating kinase 4
*NCSTN*	nicastrin
*PRNP*	prion protein gene
PCR	polymerase chain reaction
PMCA	protein misfolding cyclic amplification
SD	standard deviation
*SORL1*	sortilin-related receptor
