# Supplementary material for: First Report of the Potential Bovine Spongiform Encephalopathy (BSE)-Related Somatic Mutation E211K of the Prion Protein Gene (PRNP) in Cattle
Source: Int J Mol Sci. 2020 Jun 15;21(12):4246. doi: 10.3390/ijms21124246 (PMC7352198; doi:10.3390/ijms21124246)
Supplement: Supplementary file 1 [file ijms-21-04246-s001.pdf]

Supplementary Table S1 In-silico annotations of pathogenic *PRNP* mutations in human prion diseases

Mutations	PolyPhen-2		PANTHER	
	Score	Prediction	Preservation time	Prediction
S17G	0.528	Possibly damaging	176	Probably benign
P39L	1.000	Probably damaging	361	Possibly damaging
P102L	1.000	Probably damaging	361	Possibly damaging
P105L	1.000	Probably damaging	324	Possibly damaging
P105T	0.998	Probably damaging	324	Possibly damaging
P105S	0.997	Probably damaging	324	Possibly damaging
G114V	1.000	Probably damaging	220	Possibly damaging
A117V	0.999	Probably damaging	176	Probably benign
G131V	1.000	Probably damaging	361	Possibly damaging
D167N	0.001	Benign	220	Possibly damaging
V176G	0.998	Probably damaging	361	Possibly damaging
D178N-129M	1.000	Probably damaging	361	Possibly damaging
D178N-129V	1.000	Probably damaging	361	Possibly damaging
V180I	0.009	Benign	220	Possibly damaging
T183A	0.978	Probably damaging	324	Possibly damaging
H187R	0.989	Probably damaging	220	Possibly damaging
T188K	0.996	Probably damaging	324	Possibly damaging

E196K	0.624	Possibly damaging	220	Possibly damaging
E196A	0.472	Possibly damaging	220	Possibly damaging
F198S	0.994	Probably damaging	220	Possibly damaging
E200K	0.995	Probably damaging	361	Possibly damaging
E200G	0.994	Probably damaging	361	Possibly damaging
D202N	1.000	Probably damaging	220	Possibly damaging
V203I	0.001	Benign	176	Probably benign
R208C	1.000	Probably damaging	220	Possibly damaging
R208H	0.999	Probably damaging	220	Possibly damaging
V210I	0.803	Possibly damaging	220	Possibly damaging
E211Q	0.992	Probably damaging	220	Possibly damaging
Q212P	0.930	Possibly damaging	220	Possibly damaging
I215V	0.000	Benign	220	Possibly damaging
Q217R	0.961	Probably damaging	220	Possibly damaging
M232R	0.082	Benign	91	Probably benign
M232T	0.000	Benign	91	Probably benign
P238S	1.000	Probably damaging	361	Possibly damaging
